# Multiparametric Profiling for Identification of Chemosensitizers against Gram-Negative Bacteria

**DOI:** 10.3389/fmicb.2018.00204

**Published:** 2018-02-19

**Authors:** Vincent Lôme, Jean-Michel Brunel, Jean-Marie Pagès, Jean-Michel Bolla

**Affiliations:** ^1^UMR MD1, Aix-Marseille University, IRBA, TMCD2, Facultés de Médecine et de Pharmacie, Marseille, France; ^2^Centre de Recherche en Cancérologie de Marseille (CRCM), CNRS, UMR7258, Institut Paoli Calmettes, Aix-Marseille Université, UM 105, Inserm, U1068, Faculté de Pharmacie, Marseille, France

**Keywords:** antibiotic resistance, combination therapy, automated platform, whole-cell screening, hit-to-lead

## Abstract

Antibiotic resistance is now a worldwide therapeutic problem. Since the beginning of anti-infectious treatment bacteria have rapidly shown an incredible ability to develop and transfer resistance mechanisms. In the last decades, the design variation of pioneer bioactive molecules has strongly improved their activity and the pharmaceutical companies partly won the race against the clock. Since the 1980s, the new classes of antibiotics that emerged were mainly directed to Gram-positive bacteria. Thus, we are now facing to multidrug-resistant Gram-negative bacteria, with no therapeutic options to deal with them. These bacteria are mainly resistant because of their double membrane that conjointly impairs antibiotic accumulation and extrudes these molecules when entered. The main challenge is to allow antibiotics to cross the impermeable envelope and reach their targets. One promising solution would be to associate, in a combination therapy, a usual antibiotic with a non-antibiotic chemosensitizer. Nevertheless, for effective drug discovery, there is a prominent lack of tools required to understand the rules of permeation and accumulation into Gram-negative bacteria. By the use of a multidrug-resistant enterobacteria, we introduce a high-content screening procedure for chemosensitizers discovery by quantitative assessment of drug accumulation, alteration of barriers, and deduction of their activity profile. We assembled and analyzed a control chemicals library to perform the proof of concept. The analysis was based on real-time monitoring of the efflux alteration and measure of the influx increase in the presence of studied compounds in an automatized bio-assay. Then, synergistic activity of compounds with an antibiotic was studied and kinetic data reduction was performed which led to the calculation of a score for each barrier to be altered.

## Introduction

Historically, the activity improvement of antibiotics has been widely performed by design variation of pioneer bioactive molecules (Bush et al., 2011; Silver, 2011). Since the 1980s, new classes of antibiotics have emerged but mainly are active against Gram-positive bacteria (Lomovskaya et al., 2006). Recent target-based high-throughput screening programs along with *in silico* studies have led to identification of hits with high potentiality. Although this strategy appears attractive, the major drawback of target-based assays is that they fail to consider the membrane translocation barriers, comprising of the bacterial permeation and the efflux pump issues (Payne et al., 2006; Stavenger and Winterhalter, 2014; Tommasi et al., 2015; Zgurskaya et al., 2015). In this context, one of the greatest challenges for the design of new scaffolds of interest against Gram-negative bacteria is to promote their intrabacterial accumulation (Nikaido, 1994; Lomovskaya et al., 2006; Spellberg and Shlaes, 2014; Stavenger and Winterhalter, 2014; Zgurskaya et al., 2015). Hence, combination therapy of a usual antibiotic with a non-antibiotic chemosensitizer seems to be one of the most promising solutions (Kristiansen et al., 2007; Mazumdar et al., 2009), being able to increase antibiotics accumulation through non-specific synergy mechanisms such as permeability, enhancement, and efflux impairment (Lewis, 2013). Nevertheless, in effective drug discovery, there is a prominent lack of tools required to understand the rules of permeation (Masi et al., 2017) and accumulation into Gram-negative bacteria (Harvey et al., 2015; Schneider et al., 2017).

**FIGURE 1:**
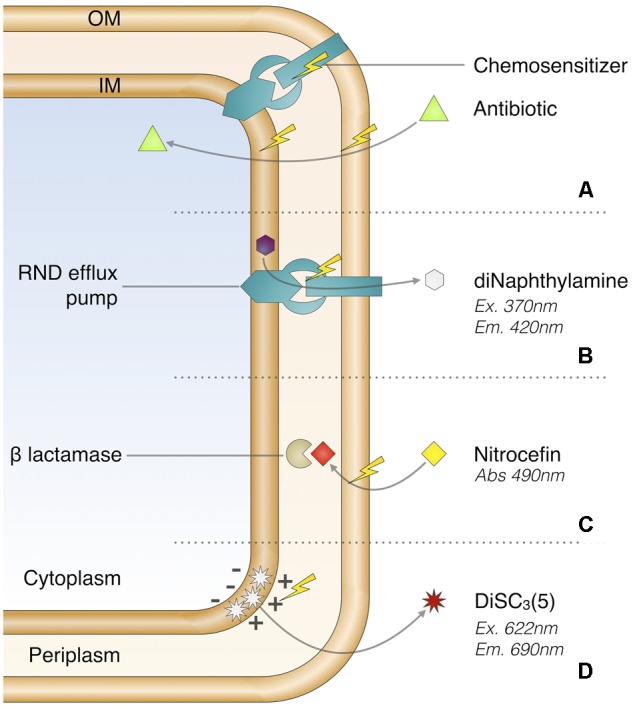
Chemosensitizer promoting drug accumulation **(A)** and real-time assays used in this study **(B–D)**. Chemosensitizer possible modes of action **(A)**, RND efflux inhibition screening **(B)**, OM permeabilization screening **(C)**, and transmembrane potential disruption assay **(D)**.

We introduce a high-content screening method for chemosensitizers discovery by quantitative assessment of drug accumulation barriers alteration and deduction of their activity profile. We adapted a series of whole-cell-based assays (**Figure 1B–D**) to the multidrug-resistant *Enterobacter aerogenes* EA289 clinical isolate since this strain presents a decreased outer membrane permeability as it does not express major porins, and an enhanced efflux transport toward antibiotics (Mallea et al., 1998; Pradel and Pagès, 2002). We assembled and analyzed a control chemicals library to perform the proof of concept.

A chemosensitizer compound can promote drug accumulation by altering at least one major cellular barrier (**Figure 1A**). Hence, the first stage of analysis was based on two ways: real-time monitoring of the RND efflux alteration using the 1,2′-diNA dye and measurement of increased influx using the nitrocefin probe in the presence of studied compounds.

In this context, kinetic data reduction was performed taking into account the biological variability among replicates, the non-specific absorbance, or fluorescence interactions between the compound and the probe, which led to the calculation of a score for each barrier to be altered.

## Materials and Methods

### Strains Used in This Study

Two bacterial strains were used in this study. The *E. aerogenes* EA289 strain is a Kan^s^ derivative of the MDR clinical isolate Ea27 (Mallea et al., 1998); the *acrB* mutant EA289Δ*acrAB* strain was constructed from the EA289 strain (Pradel and Pagès, 2002). Strains were maintained at -80°C in 15% (v/v) glycerol for cryoprotection. Bacteria were routinely grown in Cation-adjusted Mueller Hinton Broth (CAMHB) at 37°C.

### Glucose-Triggered 1,2′-Efflux Assay

A single colony of *E. aerogenes*, EA289 or EA289Δ*acrAB*, from an overnight plate, was grown in CAMHB shaking at 250 rpm at 37°C until the OD_600nm_ = 0.5 was reached. Bacteria were collected by centrifugation and re-suspended at OD_600nm_ = 0.25 in potassium phosphate buffer, K_2_HPO_4_ 20 mM, MgCl_2_ 1 mM, pH 7.0 (PPB), supplemented with the proton conductor carbonyl cyanide m-chlorophenyl-hydrazone (CCCP) 5 μM, that allows the inactivation of active efflux by de-energization of the cytoplasmic membrane (Nagano and Nikaido, 2009). Bacteria were then incubated overnight with 1,2′-dinaphthylamine (1,2′-diNA) 32 μM at 37°C. After overnight incubation, the cells were washed in PPB and aliquoted in microplates (100 μl/well) in wells pre-loaded with compounds 100 μM or controls. Membrane incorporated 1,2′-diNA was followed by monitoring the fluorescence every 30 s at 37°C (*λ*_ex_ = 370 nm; *λ*_em_ = 420 nm). Glucose 50 mM was added at 300 s to initiate bacterial energization. Plates were read on a Tecan Infinite M200 Pro multimode plate reader. Assays were performed in Greiner Bio-One 96-well plates, ref 675076 (half area, black with solid bottom). 1,2′-diNA ref D2988 was purchased from T.C.I (Zwijndrecht, Belgium) (Brunel et al., 2013).

The pipeting was performed by a Tecan Freedom EVO automated liquid handler equipped with eight fixed PTFE-coated tips, with a robotic arm to transfer plates to the Tecan Infinite M200 Pro multimode plate reader. Automation protocols were coded on Tecan EVOware and are available upon request (Tecan, Lyon, France).

### Glucose-Triggered 1,2′-diNA Efflux Assay, Kinetic Data Reduction (**Table 1**)

**Table 1:** Glucose-triggered 1,2′-diNA efflux assay, kinetic data reduction.

Kinetic transformed data	Comments
basisRFU_A_(Δ, NC)	Pre-energization fluorescence intensity of EA289Δ*acrAB* in the presence of DMSO 1%
basisRFU_A_(Δ, x)	Pre-energization fluorescence intensity of EA289Δ*acrAB* in the presence of a tested compound
areaRFU.s_B_(WT, NC)	Area under the curve after dye efflux in EA289 in the presence of DMSO 1%
finalRFU.s_B_(WT, NC)	As: areaRFU.s_B_(WT, NC) ÷ 300. Mean fluorescence intensity after dye efflux in EA289 in the presence of DMSO 1%
areaRFU.s_B_(WT, x)	Area under the curve after dye efflux in EA289 in the presence of a tested compound
finalRFU.s_B_(WT, x)	As: areaRFU.s_B_(WT, x) ÷ 300. Mean fluorescence intensity after dye efflux in EA289 in the presence of a tested compound

Kinetic profiles observed in raw data are outlined in **Supplementary Figure 1**. Typically, a-outlined curves were non-specific fluorescence controls obtained for each compound incubated with efflux deficient mutant EA289Δ*acrAB* (Δ), b were positive results for 1,2′-diNA efflux inhibition curves incubated with the wild-type (WT) strain EA289, c were control and negative results incubated with WT EA289. Quenched kinetic signals not to be analyzed were reported in **Supplementary Figure 2**, where a-outlined curves were fluorescence controls obtained incubated with EA289Δ*acrAB*, b were the same tested compounds as a-curves incubated with the WT EA289, and c were stronger quencher compounds incubated with EA289 or with EA289Δ*acrAB.*

Kinetic transformed data basisRFU, areaRFU.s were calculated systematically, from raw kinetic data and served as the basis for score calculation and quencher compounds detection. basisRFU_A_ was calculated as the mean value of the 10 first data points (A period) and represents the pre-energization fluorescence intensity [AU]; areaRFU.s_B_ was calculated as the area under the curve over data point 19 through data point 29 (B period) and represents the area under the curve of remaining 1,2′-diNA after transport [AU.t] (**Supplementary Figure 3**).

As observed in **Supplementary Figure 1a**, and consistent with the initial hypothesis, 1,2′-diNA efflux kinetics in the presence of EA289Δ*acrAB* were not sensitive to the effect of potential efflux inhibitors. However, a compound leading to the translation of EA289Δ*acrAB* efflux kinetic was considered a fluorescence quencher. The fluorescence quenching calculation is based on the comparison of the basisRFU_A_(Δ, NC) and basisRFU_A_(Δ, x) values for EA289Δ*acrAB*, in the absence and presence of tested compound, respectively.

If the value basisRFU_A_(Δ, x) < basisRFU_A_(Δ, NC) × 0.7 the fluorescence signal was considered quenched too much and a proportionality factor correction could not effectively report kinetics. Such data were not further analyzed. It is important to note that a compound having such properties on fluorescence signal is not indicative of its potential inhibitor character of efflux systems. The characterization of this activity must be pursued by other experimental methods. Otherwise, i.e., if the value basisRFU_A_(Δ, x) > basisRFU_A_(Δ, NC) × 0.7 we considered that the fluorescence signal may be corrected by proportionality factor and efflux inhibition score (%EIS) may be calculated.

The %EIS calculation comprised two steps:

(1) The finalRFU.s_B_(WT, x) values obtained for tested compounds in the presence of the WT strain were adjusted using the mean basisRFU_A_(Δ, NC) value calculated for the eight negative control (NC) replicates of the mutant strain EA289Δ*acrAB*, and the basisRFU_A_(Δ, x) values obtained for tested compounds in the presence of the mutant strain.

$$\begin{array}{c} \mathrm{finalRFU}.s_{B}{}_{,\mathrm{adjusted}}(\mathrm{WT},x)=\mathrm{finalRFU}.s_{B}(\mathrm{WT},x)\times \frac{{\mathrm{basisRFU}}_{A}(\Delta ,\mathrm{NC})}{{\mathrm{basisRFU}}_{A}(\Delta ,x)}. \end{array}$$

This step both participates in robustness of the results by correcting moderate shift in basis fluorescence observed in **Supplementary Figure 1a**, and detects strong quenchers.

(2) The mean areaRFU.s_B_(WT, NC) value calculated for the eight NC replicates of the WT strain was subtracted to the adjusted areaRFU.s_B,adjusted_(WT,x) values obtained for tested compounds in the presence of the WT strain. This step ensures results consistency between assays by correcting the biological variability among clones. The obtained values were normalized using the maximum efflux of the WT strain which was calculated by subtracting the mean basisRFU_A_(WT, x) value calculated for the eight NC replicates multiplied by the duration of the **A** period (300 s) to the mean areaRFU.s_B_(WT, NC) value calculated for the eight NC replicates of the WT strain. Finally, the %EIS was obtained as:

$$\begin{array}{c} \%\mathrm{EIS}(x)=\frac{\mathrm{finalRFU}.s_{B,\mathrm{adjusted}}(\mathrm{WT},x)-\mathrm{finalRFU}.s_{B}(\mathrm{WT},\mathrm{NC})}{{\mathrm{basisRFU}}_{A}(\mathrm{WT},\mathrm{NC})-\mathrm{finalRFU}.s_{B}(\mathrm{WT},\mathrm{NC})}\times 100. \end{array}$$

The *Z*′-factor (Zhang et al., 1999) was calculated based on the average of four screens, using the EA289Δ*acrAB* strain incubated with 1% DMSO as a positive control (PC) and the WT strain incubated with 1% DMSO as NC, in the calculation.

Kinetic data transformation, score calculation, and false positive detection were programmed and performed automatically by Tecan Magellan software. Both data from Magellan and pipetting instructions from EVOware were sent to a custom LIMS (Modul-Bio, Marseille, France), which matched results to compound names.

### Outer Membrane Permeability Assay

A single colony of *E. aerogenes*, EA289, from an overnight plate, was grown in CAMHB shaking at 250 rpm at 37°C until the OD_600nm_ = 0.5 was reached. Bacteria were collected by centrifugation and were re-suspended at OD_600nm_ = 0.25 in PPB, supplemented with CCCP 5 μM. Bacteria were mixed with nitrocefin 50 μg ml^-1^ before addition of compounds 100 μM. Nitrocefin hydrolysis was followed by monitoring the absorbance (*λ*_abs_ = 490 nm). Cell suspension was added at 100 μl per well and the absorbance read every 30 s at 37°C. Assays were performed in Greiner Bio-One 96-well plates, ref 675101 (half area, clear with flat bottom). Nitrocefin SR0112C was purchased from Oxoid (Basingstoke, United Kingdom) (Matsumoto et al., 2011).

The pipetting was performed by a Tecan Freedom EVO automated liquid handler equipped with eight fixed PTFE-coated tips, with a robotic arm to transfer plates to the Tecan Infinite M200 Pro multimode plate reader. Automation protocols were coded on Tecan EVOware and are available upon request.

### Outer Membrane Permeability Assay, Kinetic Data Reduction (**Table 2**)

**Table 2:** Outer membrane permeability assay, kinetic data reduction.

Kinetic transformed data	Comments
basisOD_A_(x)	OD value at origin in the presence of a tested compound
maxSlopeOD/hr_B_(x)	Initial slope in the presence of a tested compound
areaOD.s_C_(x)	Area under the curve in the presence of a tested compound
areaOD.s_C_(NC)	Area under the curve in the presence of DMSO 1%
maxOD_D_(PC)	Maximum OD for the PC

Kinetic profiles observed within raw data are outlined in **Supplementary Figure 4**. Typically, a-outlined curves were positive results, b were NC and true negatives, and *Y*-values at origin read at position c corresponded to the absorbance of the bacterial suspension. Biased kinetic signals were reported in **Supplementary Figure 5**, where a-outlined curves were false positives, b were associated with compounds competitively hydrolyzed with the nitrocefin β-lactam (compounds belonging to β-lactam antibiotics or β-lactamase inhibitors, families). *Y*-values at origin read at position c were expected to be 0.25 the OD_600nm_ for bacterial suspension, whereas d and e corresponded to higher OD_600nm_ due to non-specific compound absorbance at the same wavelength as the peak absorbance of hydrolyzed nitrocefin. False positive detection can be anticipated by naked eye observation of the plate during pipetting, concerned wells appearing red.

Kinetic transformed data basisOD, maxSlopeOD/hr, areaOD.s, and maxOD were calculated systematically, from raw kinetic data and served as the basis for score calculation and false positive detection. basisOD_A_ was calculated as the mean value of the five first data points (A period) and represents the *Y*-axis value at origin [OD], maxSlopeOD/hr_B_ was calculated as the maximum slope using three consecutive data points within the 10 first data points (B period) and represents the mean slope at origin [OD/t], areaOD.s_C_ was calculated as the area under the curve over the 80 first data points (C period) [OD.t], maxOD_D_(PC) was calculated as the maximum OD value using five consecutive data points over data point 71 through data point 81 (D period) in the presence of sodium dodecyl sulfate (SDS) 0.5% as the PC condition (**Supplementary Figure 6**).

Possible false positives showed kinetic curve starts that were parallel to the NC one (**Supplementary Figure 5a**). Data can be corrected by translation of the curve to the NC one, and data may be saved. The basisOD_A_(x) value can be significantly greater than what is observed with the NC both in the case of a true positive or in the case of false positives (**Supplementary Figures 4a**, and **5a**, respectively). The distinction between true and false positives can be done through their initial slope maxSlopeOD/hr_B_(x): false positives showed an initial slope that was similar to the NC one, whereas true positives showed a significantly greater one. According to repeated observations the threshold value for maxSlopeOD/hr_B_(x) was arbitrarily set to 1 OD hr^-1^.

If maxSlopeOD/hr_B_(x) < 1 OD hr^-1^, the initial slope was considered to be close to the NC one. The translation may be then applied within outer membrane permeabilization score (%OPS) calculation. Otherwise, i.e., if maxSlopeOD/hr_B_(x) > 1 OD hr^-1^, the initial slope was considered significant, due to rapid hydrolytic reaction start, before reading has taken place. It is a specific absorbance of hydrolyzed nitrocefin, and translation must therefore not be applied.

The %OPS calculation comprised two steps:

(1) All curves were translated so their *Y*-axis value at origin equals 0, by subtracting the _basisOD_A_(x)_ value multiplied by the duration of the C period (2400 s). This step both corrects false positives observed in **Supplementary Figure 5a**, and participates in robustness of the result by overcoming any differences in optical path length between well (optical meniscus may not be identical for all wells, even after the agitation of the plate). The differences in endpoint value may be infinitesimal but they are exacerbated by the area under the curve calculation and can alter the further ranking of low activity compounds. This calculation was performed systematically except when a rapid acting true positive was detected. Therefore, such curves did not benefit the subtraction of basisOD_A_(x) × 2400 adjustment nonetheless optical path variability did not interfere with rapid acting true positives ranking.

$$\begin{array}{c} \mathrm{areaOD}.s_{C,\mathrm{adjusted}}(x)=\mathrm{areaOD}.s_{C}(x)-(\mathrm{basisODA}(x)\times 2400). \end{array}$$

(2) The mean areaOD.s_C,adjusted_(NC) value calculated for the eight NC replicates was subtracted to the mean areaOD.s_C,adjusted_(x) value calculated for the two replicates of each tested compound. This step ensures results consistency between assays by correcting the biological variability among clones.

Within the same calculation step, obtained values were normalized, using the PC condition corresponding to bacterial cells lysis in the presence of SDS 0.5%. The mean maxOD_D_(PC) value was calculated for the eight PC replicates, and the %OPS was calculated as:

$$\begin{array}{c} \%\mathrm{OPS}(x)=\frac{\mathrm{areaOD}.s_{C,\mathrm{adjusted}}(x)-\mathrm{areaOD}.s_{C,\mathrm{adjusted}}(\mathrm{NC})}{\left({\mathrm{maxOD}}_{D}(\mathrm{PC})\times 2400)-\mathrm{areaOD}.s_{C}(\mathrm{NC}\right)}\times 100. \end{array}$$

The *Z*′-factor (Zhang et al., 1999) was calculated based on the average of two screens, using chlorhexidine 100 μM as PC and 1% DMSO as NC, in the calculation.

Kinetic data transformation, score calculation, and false positive detection were programmed and performed automatically by Tecan Magellan software. Both data from Magellan and pipetting instructions from EVOware were sent to a custom LIMS (Modul-Bio, Marseille, France), which matched results to compound names.

### Transmembrane Potential Disruption

A single colony of *E. aerogenes*, EA289, from an overnight plate, was grown in CAMHB shaking at 250 rpm at 37°C until the OD_600nm_ = 0.5 was reached. Bacteria were collected by centrifugation and were re-suspended at OD_600nm_ = 0.25 in HEPES 5 mM, EDTA 10 mM, pH 7.0, and then washed in HEPES 5 mM, pH 7.0 with 3-3′-dipropylthiadicarbocyanine iodide (diSC_3_(5)) 8 μM. The membrane potential-sensitive cyanine dye diSC_3_(5) distributes between cells and the medium depending on the cytoplasmic membrane potential gradient. Released diSC_3_(5) was quantified by measuring the fluorescence (*λ*_ex_ = 622 nm; *λ*_em_ = 690 nm) 300 s after the addition of compounds 100 μM. A control experiment was performed for every tested compound where the cells were treated with chlorhexidine 300 μM as lysis solution, to normalize the results. Cell suspension was added at 100 μl/well and the fluorescence read every 30 s at 37°C. Plates were read on a Tecan Infinite M200 Pro multimode plate reader. Assays were performed in Greiner Bio-One 96-well plates, ref 675076 (half area, black with solid bottom). 3,3′-dipropylthiadicarbocyanine iodide (diSC_3_(5)) ref 84923 was purchased from Anaspec Inc. (Fremont, CA, United States).

The pipetting was performed by a Tecan Freedom EVO automated liquid handler equipped with eight fixed PTFE-coated tips, with a robotic arm to transfer plates to the Tecan Infinite M200 Pro multimode plate reader. Automation protocols were coded on Tecan EVOware and are available upon request.

Results are summarized in **Supplementary Table 2**. Typically, the average fluorescence intensity after the addition of compounds was between 1000 and 3000 A.U. for NC (incubated with DMSO 1%) and negative results, and between 4000 and 10,000 A.U. for positive results. The PC condition included compounds with the lysis solution and showed fluorescence intensities between 45,000 and 60,000 A.U.

### Monodose Chemosensitization Assay (**Table 3**)

**Table 3:** Monodose Chemosensitization assay, kinetic data reduction.

Endpoint measurement	Comments
OD(x)	OD value for the growth of EA289 in the presence of a tested compound
OD(background)	OD value for sterile CAMHB in the presence of 0.1% DMSO
OD(NC)	OD value for the growth of EA289 in the presence of 0.1% DMSO

Previously selected hits from the control chemicals library were tested at 10 μM for 48 h growth inhibition of *E. aerogenes* EA289. Compounds were tested in both conditions: in combination with doxycycline to assess combinatory growth inhibition and alone to assess direct growth inhibition. The screening protocol was performed according to Ejim et al.’s (2011) antibacterial combination screening, with several adjustments (Ejim et al., 2011). Screening was carried out in 200 μl in 96-well F-bottom clear plates, in duplicate, using CAMHB with 0.1% DMSO and a library compound concentration of 10 μM. When used, the concentration of doxycycline was 5 μg ml^-1^, corresponding to half minimal inhibitory concentration (MIC) value obtained under the same conditions of the monodose chemosensitization assay. The preliminary MIC assay was performed in duplicate using five shifted concentration gradients of doxycycline, from 100 to 60 μg ml^-1^. The 48 h MIC value obtained for *E. aerogenes* EA289 was 10 μg ml^-1^. The inoculum of approximately 5 × 10^5^ CFU ml^-1^ was prepared from an overnight plate. A single colony of *E. aerogenes*, EA289, was grown in CAMHB shaking at 250 rpm at 37°C until the OD_600nm_ = 0.5 was reached. Background controls (eight wells per plate) contained only media and DMSO (these were also the sterility controls). Growth controls, also eight wells per plate, contained media, DMSO, and inoculum. Plates were incubated at 37°C for 48 h. The pipetting was performed by a Tecan Freedom EVO automated liquid handler equipped with eight fixed PTFE-coated tips, with a robotic arm to transfer plates to the Tecan Infinite M200 Pro multimode plate reader. Plates were read at 600 nm after 5 min shaking. Automation protocols were coded on Tecan EVOware and are available upon request.

For each test well, the percentage growth and the percentage growth inhibition were calculated as:

$$\begin{array}{c} \%\mathrm{Growth}(x)=\frac{\mathrm{OD}(x)-\mathrm{OD}(\mathrm{background})}{\mathrm{OD}(\mathrm{NC})-\mathrm{OD}(\mathrm{background})}\times 100 \end{array}$$

$$\begin{array}{c} \%\mathrm{Growth}\mathrm{inhibition}=100-\%\mathrm{Growth}(x). \end{array}$$

The data set consisted in eight replicates in the absence of antibiotic and eight replicates in the presence of doxycycline. Results were represented as a *XY*-chart showing the mean value of eight replicates for combinatory growth inhibition percentage, in the presence of doxycycline (+DOX) as *Y*-value, and the mean value of eight replicates for direct growth inhibition percentage in the absence of antibiotic (-DOX) as *X*-value (**Figure 4**).

Finally, the monodose chemosesitizer score (%MCS) was calculated as:

$$\begin{array}{c} \%\mathrm{MCS}(x)=\%\mathrm{Growth}\mathrm{inhibition}{\left(x\right)}_{+DOX}-\%\mathrm{Growth}\mathrm{inhibition}{\left(x\right)}_{-\mathrm{DOX}}. \end{array}$$

Compounds showing %MCS > 40% were considered lead chemosensitizers. Results are shown in **Figure 4**.

The *Z*′-factor (Zhang et al., 1999) was calculated based on the average of eight screens, using NV845 10 μM as PC and tetracycline 10 μM as NC, in the calculation.

Hits showing 100% growth inhibition in the absence of antibiotic may be retested at one-tenth or one-hundredth the concentration, i.e., 1 or 0.1 μM, as it indicates such hit was tested above its MIC value. Results are shown in **Supplementary Figure 7**.

Score calculation was programmed and performed automatically by Tecan Magellan software. Both data from Magellan and pipetting instructions from EVOware were sent to a custom LIMS (Modul-Bio, Marseille, France), which matched results to compound names.

## Results

**FIGURE 2:**
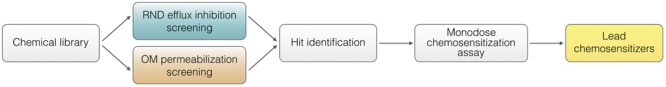
Chemosensitizer discovery workflow.

In order to demonstrate the efficiency of this multiparametric profiling method as a tool for chemosensitizer discovery, an 80 control compounds library was assembled and tested, including: reference permeabilizers, antibiotics, together with natural or synthetic molecules (**Supplementary Table 1**). The first stage of analysis consisted in identifying hit compounds against EA289 along with understanding their activity profile (**Figure 2**). These assays were performed by using a multimode plate reader linked to an automated liquid handler in order to minimize exposure time variation of the tested compounds to bacteria, human error, and the pipetting precision issues. We determined that a 100 μM dose provided the most discriminatory dynamic range to screen compounds on real-time assays. Growth and buffer conditions were also homogenized among the assays to obtain consistent data for profiling purposes.

### Efflux Inhibition Screening

**FIGURE 3:**
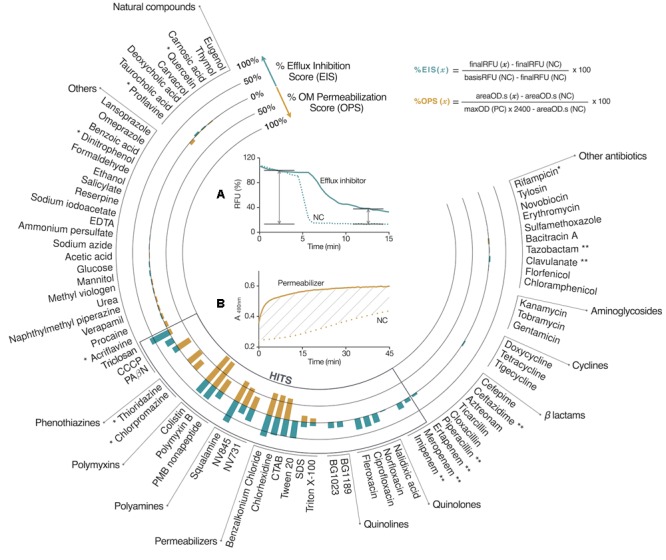
Hit identification and profiling. The chemical library was tested at 100 μM on EA289. Compounds showing a score exceeding 10% on one tested activity were considered hit compounds. Hits showing significant %OPS attributed the permeabilizer profile. The anti-efflux profile was associated with hits showing %EIS-only. Data reduction for RND efflux inhibition screening **(A)** and for OM permeabilization screening **(B)**. ^∗^, Efflux inhibition data resulted in 30% or more fluorescence quenching, data were not considered. ^∗∗^, %OPS was a negative value, due to β-lactamase inhibitors, or β-lactam competition with nitrocefin hydrolysis. NV845, {3-[(3-amino-propyl)-methyl-amino]-propyl}-((2E,6E)-3,7,11-trimethyl-dodeca-2,6,10-trienyl)-amine; NV731, {3-[bis-(3-amino-propyl)-amino]-propyl}-(3,7-dimethyl-octa-2,6-dienyl)-amine; BG1023, N1-(7-chloroquinolin-4-yl)-N2,N2-diisopropylethane-1,2-diamine; BG1189, 3-(3-dimethylamino-propyl)-6-nitro-3H-quinazolin-4-one.

The effect on RND efflux was assessed using the 1,2′-diNA fluorescent dye, a substrate of the AcrAB–TolC pump (**Figure 1B**) which is fluorescent when loaded into the bacteria (Bohnert et al., 2011). The dye efflux is then triggered by glucose addition as energy source causing the fluorescence decrease. The plate layout included each compound to be tested both against EA289 and EA289Δ*acrAB*, which was included as a control since it is unable to efflux 1,2′-diNA (**Supplementary Figures 1a, 3**). To prevent biological variability among replicates, and to adjust the overall fluorescence change due to the tested compound, basis fluorescence, i.e., before energization, was calibrated for each compound using the mean fluorescence obtained with EA289Δ*acrAB*. The efflux inhibition score (%EIS) was calculated (**Figure 3A**) by subtracting the final fluorescence, i.e., at the end of the measurement, obtained in the absence of compound from the value obtained in the presence of the tested compound. %EIS accounts for the proportion of retained 1,2′-diNA over the efflux capacity of EA289 without compound. Practically, a total retention of the dye within the membrane as observed with the *acrAB* mutant corresponded to a 100% EIS whereas a 0% EIS was related to fully functional efflux pumps. The data analysis included systematic signal adjustments and score calculation resulting in a robust *Z*′-factor (Zhang et al., 1999): 0.71. Thus, we determined that a score exceeding 10% was related to a significant real-time effect, leading to compound selection as a hit. From this step of screening, among the tested library, the following compounds showed a significant %EIS: triclosan, CCCP, PAßN, polymyxins, polyamines, permeabilizers, quinolines, fluoroquinolones, imipenem, and meropenem.

### Membrane Permeabilization Screening

Membrane permeability was assessed using the measurement of nitrocefin hydrolysis by periplasmic β-lactamases (**Figure 1C**). In this case, the outer membrane permeabilization score (%OPS) was calculated (**Figure 3B**) by subtracting the basal hydrolysis area under the curve due to passive transport of nitrocefin from the area obtained in the presence of the tested compound, in order to prevent biological variability between replicates. This analysis led to robust data as it takes into account both the initial slope, the maximum plateau, and the mean absorbance, of each curve. Finally, a bacterial lysis presenting a maximum β-lactamase hydrolysis rate was associated to a 100% OPS and a 0% OPS was related to a basal hydrolysis rate (background). The data analysis included systematic signal adjustments and score calculations resulting in a robust *Z*′-factor (Zhang et al., 1999): 0.78. Thus, we determined that a score exceeding 10% was related to a significant real-time effect, leading to compound selection as a hit. From this step of screening, among the tested library, the following compounds showed a significant %OPS: triclosan, PAßN, polymyxins, polyamines, benzalkonium chloride, chlorhexidine, CTAB, SDS, and Triton X-100. However, since nitrocefin is a good substrate for AcrAB–TolC efflux system (Nagano and Nikaido, 2009), we cannot exclude the possibility that the apparent permeability enhancement was caused by the inhibition of efflux, for example in the case of PAßN.

### Transmembrane Potential Assay

As proton-motive force drives RND efflux pumps, disruption of transmembrane potential could lead to efflux impairment. In this context, it was critical to understand if an observed %EIS resulted in the inner membrane potential disruption or by specifically targeting efflux systems. This parameter was assessed with membrane potential sensitive diSC_3_(5) probe that becomes fluorescent once released into the external medium following the disruption of the membrane potential (Katayama et al., 2007; **Figure 1D**). Despite our efforts, the robustness of this assay was strongly impaired by repeated quenching effect. Under this consideration, this experiment was applied as a yes/no type of assay to provide supplementary information about the mechanism of action of the considered hit (**Supplementary Table 1**). The following hits showed a significant transmembrane potential disruption effect: triclosan, polymyxins, squalamine, permeabilizers, and quinolines.

### Monodose Chemosensitization Screening

The second stage of the approach consisted in investigating if the observed barrier alteration by a hit compound led to effective chemosensitization. For this purpose, selected hits were tested for growth inhibition of EA289, both alone, and in combination with an intrabacterial antibiotic. Doxycycline was selected as a probe to assess chemosensitization in EA289 since it has been previously demonstrated that, besides target-based resistance mechanisms, acquired resistance to tetracyclines in Enterobacteriaceae was due to efflux pumps overproduction and to reduce outer membrane permeability (Thaker et al., 2009; Bohnert et al., 2010). We hypothezised that an effective chemosensitizer significantly increases growth inhibition when combined with this antibiotic.

We observed that a 10 μM dose provided the most discriminatory dynamic range on growth inhibition assays. Doxycycline was combined at a 5 μg ml^-1^ concentration corresponding to one half the MIC against EA289. Under these conditions, the monodose chemosensitization score (%MCS) was calculated by comparing growth inhibition by the compound alone and when combined with the antibiotic. In this context, *Z*′-factor calculation for %MCS led to a 0.58 value, implying a more stringent selection to lead chemosensitizers, which required a %MCS exceeding 40%. Finally, identified lead chemosensitisers were NV731, NV845, PAßN, and PMB nonapeptide.

## Discussion

The approach presented herein, resulted in highly reproducible data, detected and efficiently profiled 24 hits (**Supplementary Figure 8**). Taking into account our data, we were able to discriminate four activity profiles:

• Non-specific permeabilizers showing both a significant outer membrane permeabilization activity and an efflux inhibition one, that was related to transmembrane potential disruption; including: polymyxines (Vaara, 1992), squalamine (Di Pasquale et al., 2010), and permeabilizers. We could expect compounds within this profile present low cytotoxicity as they were not specifically acting, and their use might be relevant as desinfectants. However, polymyxin and squalamine present a low cytotoxicity against eukaryotic cells, and their human therapeutic use is well established (Pogue et al., 2017).
• Specific dual-effect hits showed both a significant outer membrane permeabilization activity and an efflux inhibition one, that was independent from transmembrane potential disruption; including: PAßN, NV845, and NV731. As data will be accumulated, it would be of particular interest to discern which moiety or which chemical property participates in which activity. As new prospective strategies are currently developed to associate two molecules with compatible activities, one can consider the independent development of two associated properties, before linkage of both partners to build hybrid active molecules (Gorityala et al., 2016).
• Specific efflux pump inhibition hits showed a significant efflux inhibition activity alone, including: fluoroquinolones, imipenem, and meropenem antibiotics. Although we hypothesized that antibiotics inhibited efflux by competition with the 1,2′-diNA dye, inhibitors by direct interaction may aggregate within this profile. The possible direct interactions may include binding with TolC causing to block the exit duct (Gilardi et al., 2017), covalent binding with AcrB causing to alter the conformational change of the three AcrB protomers therefore blocking the pump, direct interaction with AcrA causing the disruption of the AcrAB–TolC complex (Daury et al., 2016).
• Finally, non-specific efflux inhibition hits, that was related to transmembrane potential disruption, include triclosan and quinolines. Due to quenching effect, we were not able to reproduce the well-known proton gradient uncoupling effect of CCCP, but we expected it to aggregate within this profile (Venter et al., 2015).

A hit-compound selected after stage 1 was either able to permeabilize outer-membrane (by letting the β-lactam nitrocefin enter the periplasm), or inhibit AcrAB-mediated efflux. Hits showing a significant %EIS with no significant %OPS were grouped as anti-efflux whereas all other hits were grouped as permeabilizers. As both barriers have previously been demonstrated to be resistance machanisms against many classes of antibiotics (Masi et al., 2017), one antibiotic was used to measure for the chemosensitzer activity. However, we cannot exclude this approach may select for doxycycline-specific chemosensitizers along with regular chemosensitizers.

**FIGURE 4:**
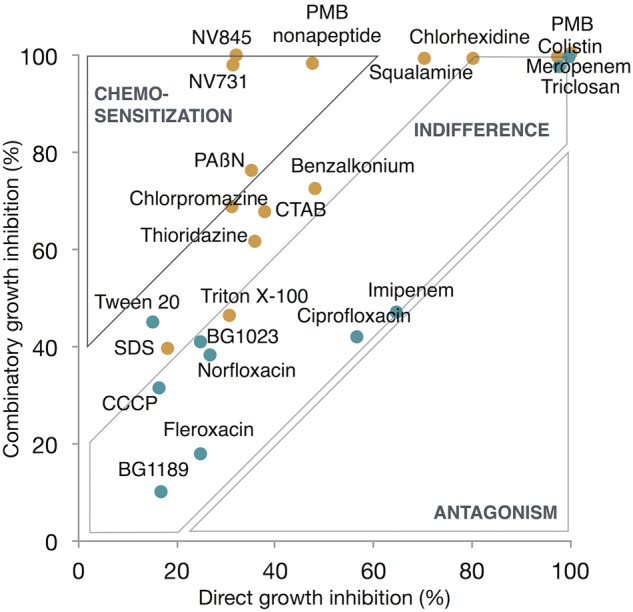
Monodose chemosensitization assay. Anti-efflux-profiled hits (blue dots), permeabilizer-profiled hits (orange dots), were tested at 10 μM for 48 h growth inhibition of EA289. Compounds were tested in both conditions: in combination with doxycycline to assess combinatory growth inhibition and alone to assess direct growth inhibition. Compounds showing 40% or more inhibition when combined with the intrabacterial targeted antibiotic were considered lead chemosensitizers.

In order to link barrier to accumulation alteration and effective chemosensitization, hits were further tested in a monodose chemosensitization assay. Although most of the lead chemosensitizers were found to be permeabilizer-profiled (**Figure 4**), we aimed to quantify the contribution of each barrier to be altered. From the data set composed of MCS, OPS, and EIS, a multiple linear regression analysis was performed. We hypothesized that %MCS was dependant on %OPS or %EIS and performed a multiple linear regression analysis which resulted in the following equation %MCS = 0.3 × %OPS + 0.2 × %EIS linking chemosensitization with the barriers to be impaired (**Supplementary Table 3**). As there was a very high chemical diversity, a wide variety of activities (efflux inhibitors, permeabilizers, dual effects, etc.), and too few data points, the statistical model remains to be improved, and the formula will be refined with the accumulation of further hits. However, the multiple linear regression model as presented has the purpose: to help define the best strategy for chemosensitization. At this point, the model tends to show the best strategy would be to develop a dual-effect chemosensitizer. As a part of broader screening campaigns this model will be refined and quantitatively respond more reliably to the relative importance of the two barriers in resistance. In the long run, this model opens two perspectives. On the one hand, it will help quantifying the contribution of each barrier in the overall resistance to antibiotics. On the other hand, this equation may be a drug-design tool that will describe the objectives to build the best antibiotic adjuvant; along with the results of QSAR (structure vs. %OPS and structure vs. %EIS) pharmacophores will be identified.

In summary, this method originally complements current bacteriological assays as it provides new tools for quantitative structure–activity relationships in the development of new chemosensitizers against Gram-negative pathogens (Lewis, 2013; Schneider et al., 2017). Thus, this method could be applied in the early search for new molecules, prior to usual studies such as checkerboard assays including several bacterial species and combined with several intrabacterial antibiotics. Besides the screening purpose, we expect the accumulated data through the scoring tools developed therin will help elucidate the rules for Gram-negative bacteria cell entry and drug accumulation (Payne et al., 2006; Silver, 2008).

## Author Contributions

All authors conceived the approach. VL developed and performed the high-throughput assays and developed the data analysis methods. VL, J-MBr, and J-MBo wrote the main manuscript text. VL prepared the figures. All authors reviewed the manuscript.

## Conflict of Interest Statement

The authors declare that the research was conducted in the absence of any commercial or financial relationships that could be construed as a potential conflict of interest.

## Supplementary Material

FIGURE S1 Example raw data from an experiment plate, obtained for the RND Efflux inhibition screening. Compounds incubated with Ea289ΔacrAB (Δ) **(a)**, compounds incubated with Ea289 **(b,c)**.

FIGURE S2 Example raw data from an experiment plate, obtained for the RND Efflux inhibition screening. Compounds incubated with Ea289ΔacrAB (Δ) **(a)**, compounds incubated with Ea289 **(b)**.

FIGURE S3 RND Efflux inhibition kinetic data reduction. Data reduction for diNaphthylamine real-time efflux. Kinetics were selected from example raw data: NV845 as test and 1% DMSO as negative control conditions.

FIGURE S4 Example raw data from one experiment plate, obtained for the outer membrane permeability screening.

FIGURE S5 Example raw data from one experiment plate, obtained for the outer membrane permeability screening.

FIGURE S6 Outer membrane permeabilization kinetic data reduction. Data reduction for outer membrane permeabilization screening. Kinetics were selected from example raw data: benzalkonium chloride as test and 1% DMSO as negative control conditions.

FIGURE S7 Monodose Chemosensitization retest. Above-MIC compounds were re-tested at 1 & 0.1μM.

FIGURE S8 Validation of control chemicals mode of action. Real-time assays results for selectied hits were evaluated in a binary.

TABLE S1 Components of the control chemicals library.

TABLE S2 Transmembrane potential disruption assay. Showed transmembrane potential disruption (+), did not show transmembrane potential disruption (-), the measurement could not be assessed due to strong fluorescence quenching (q).

TABLE S3 Relationship between %OPS, %EIS and %MCS by linear regression analysis. The relationship between chemosensitization and barriers to be altered was modeled in a multiple linear regression analysis. %MCS was hypothesized to be dependent on %OPS or %EIS and the analysis was performed on the previously selected hits except thioridazine and chlorpromazine as EIS could not be calculated. The full data set comprised %EIS, %OPS and %MCS for the 22 selected compounds. The MCS was calculated after retest at lower concentration as indicated previously for colistin, polymyxin B, meropenem and triclosan.
