# Supplementary figures and images for: Multiparametric Profiling for Identification of Chemosensitizers against Gram-Negative Bacteria
Source: Front Microbiol. 2018 Feb 19;9:204. doi: 10.3389/fmicb.2018.00204 (PMC5845390; doi:10.3389/fmicb.2018.00204)

# Fluorescence Intensity (A.U.)

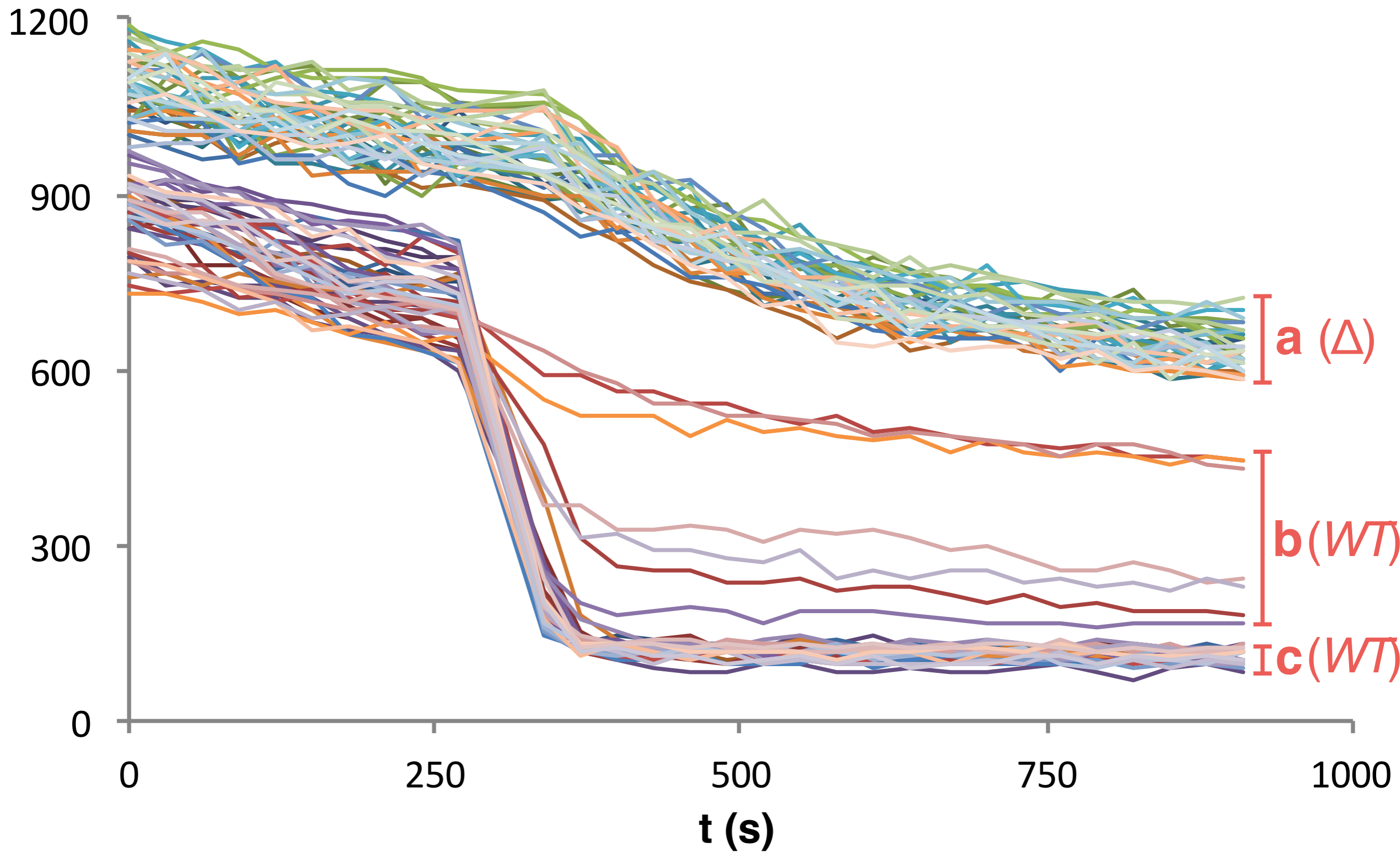

Supplement: FIGURE S1 — Example raw data from an experiment plate, obtained for the RND Efflux inhibition screening. Compounds incubated with Ea289ΔacrAB (Δ) (a), compounds incubated with Ea289 (b,c). [file Image_1.PDF]

# Fluorescence Intensity (A.U.)

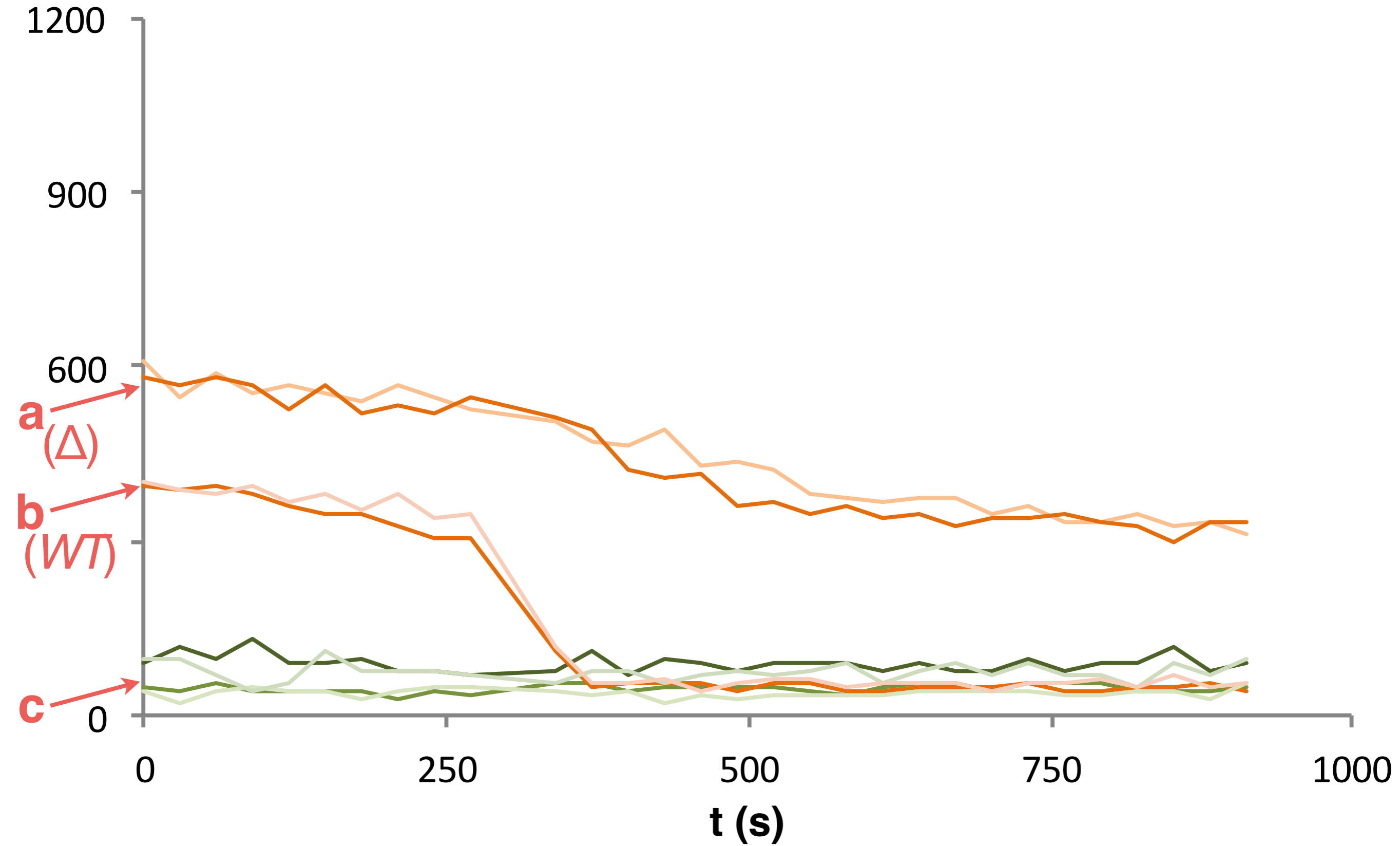

Supplement: FIGURE S2 — Example raw data from an experiment plate, obtained for the RND Efflux inhibition screening. Compounds incubated with Ea289ΔacrAB (Δ) (a), compounds incubated with Ea289 (b). [file Image_2.PDF]

# Fluorescence Intensity (A.U.)

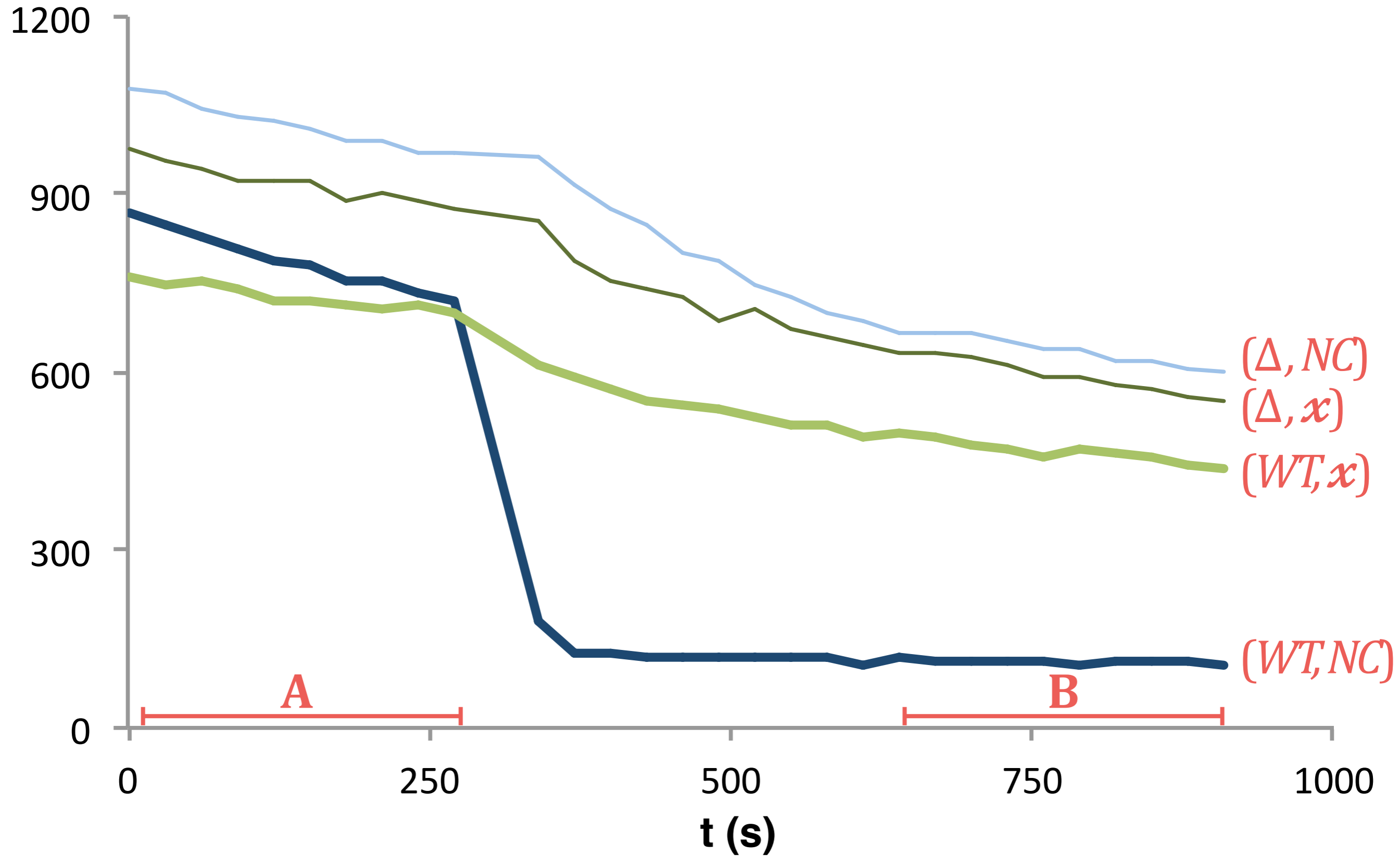

Supplement: FIGURE S3 — RND Efflux inhibition kinetic data reduction. Data reduction for diNaphthylamine real-time efflux. Kinetics were selected from example raw data: NV845 as test and 1% DMSO as negative control conditions. [file Image_3.PDF]

**OD (490 nm)**

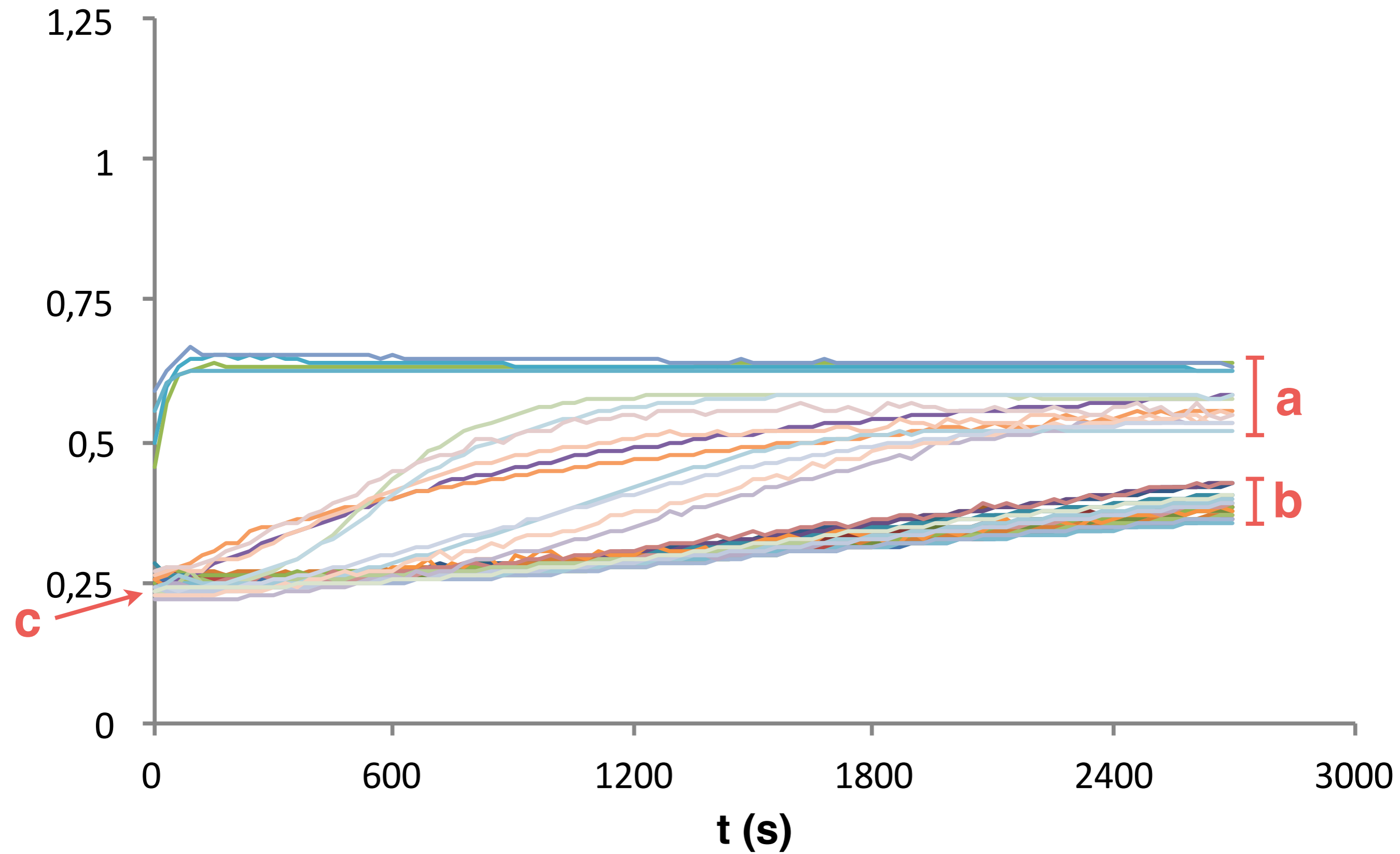

Supplement: FIGURE S4 — Example raw data from one experiment plate, obtained for the outer membrane permeability screening. [file Image_4.PDF]

**OD (490 nm)**

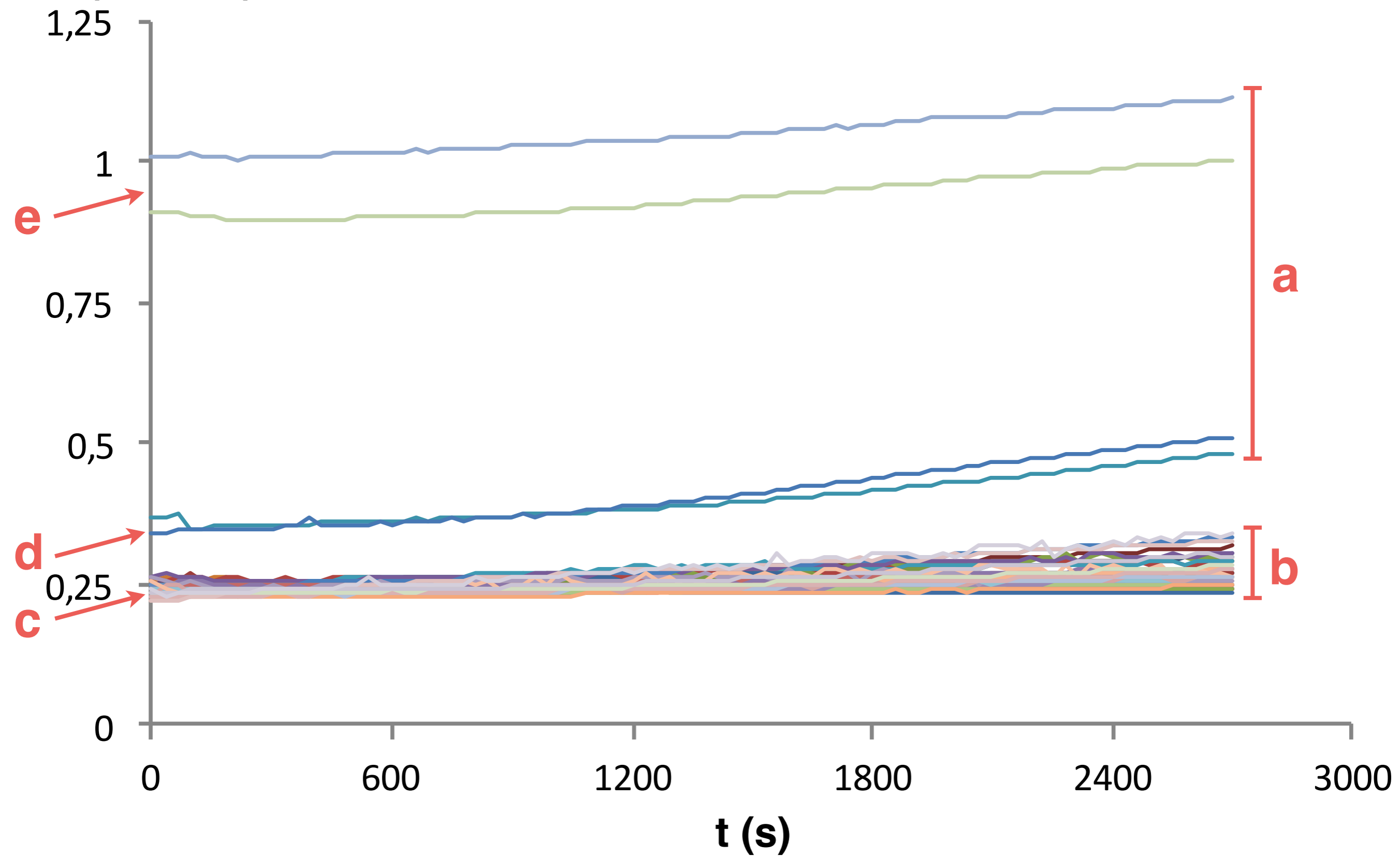

Supplement: FIGURE S5 — Example raw data from one experiment plate, obtained for the outer membrane permeability screening. [file Image_5.PDF]

OD (490 nm)

0,75

0,5

0,25

0

$\text{maxSlopeOD}/\text{hr}_B(x)$

$(x)$

$(NC)$

A

B

C

D

0

600

1200

1800

2400

3000

t (s)

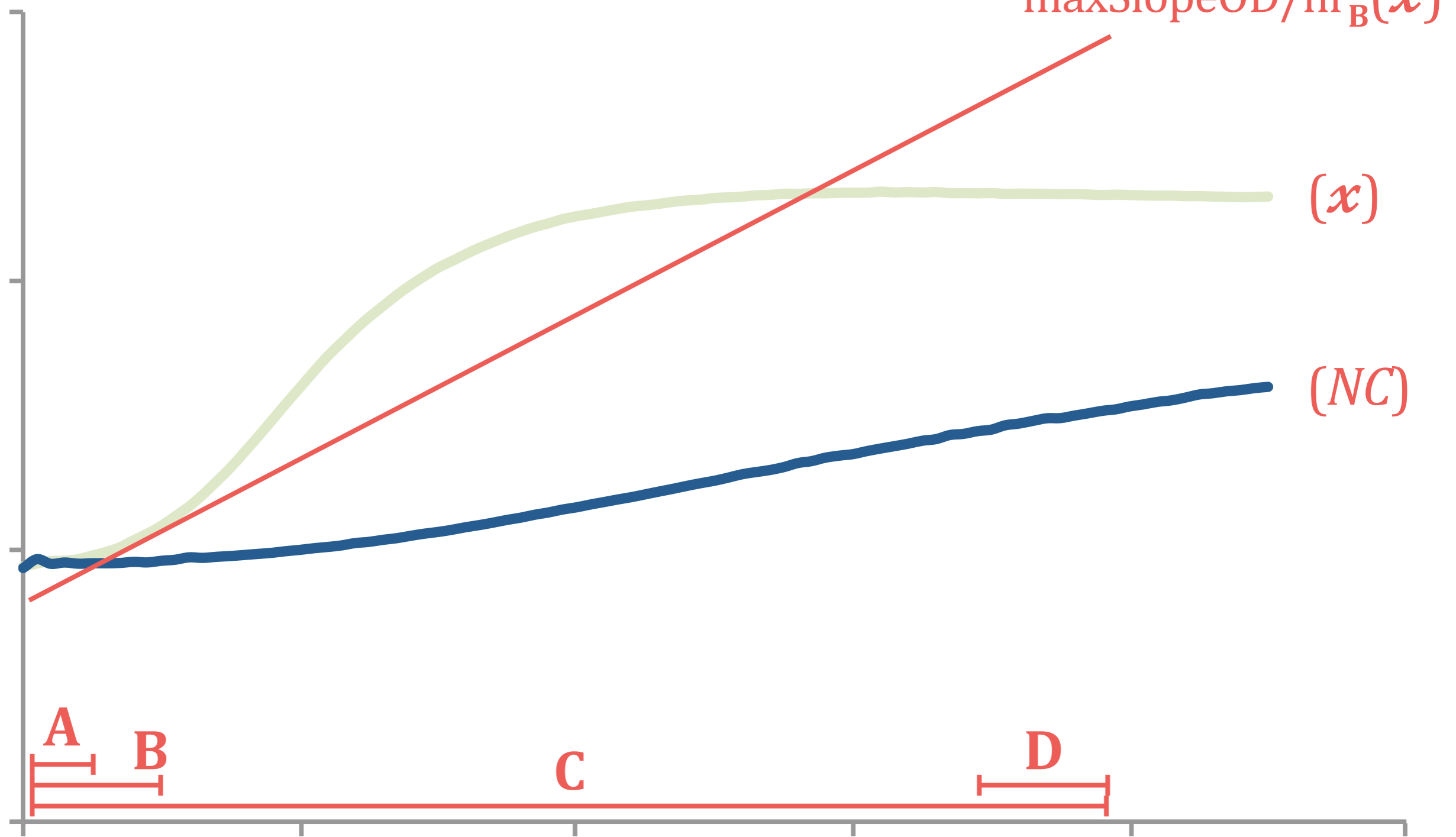

Supplement: FIGURE S6 — Outer membrane permeabilization kinetic data reduction. Data reduction for outer membrane permeabilization screening. Kinetics were selected from example raw data: benzalkonium chloride as test and 1% DMSO as negative control conditions. [file Image_6.PDF]

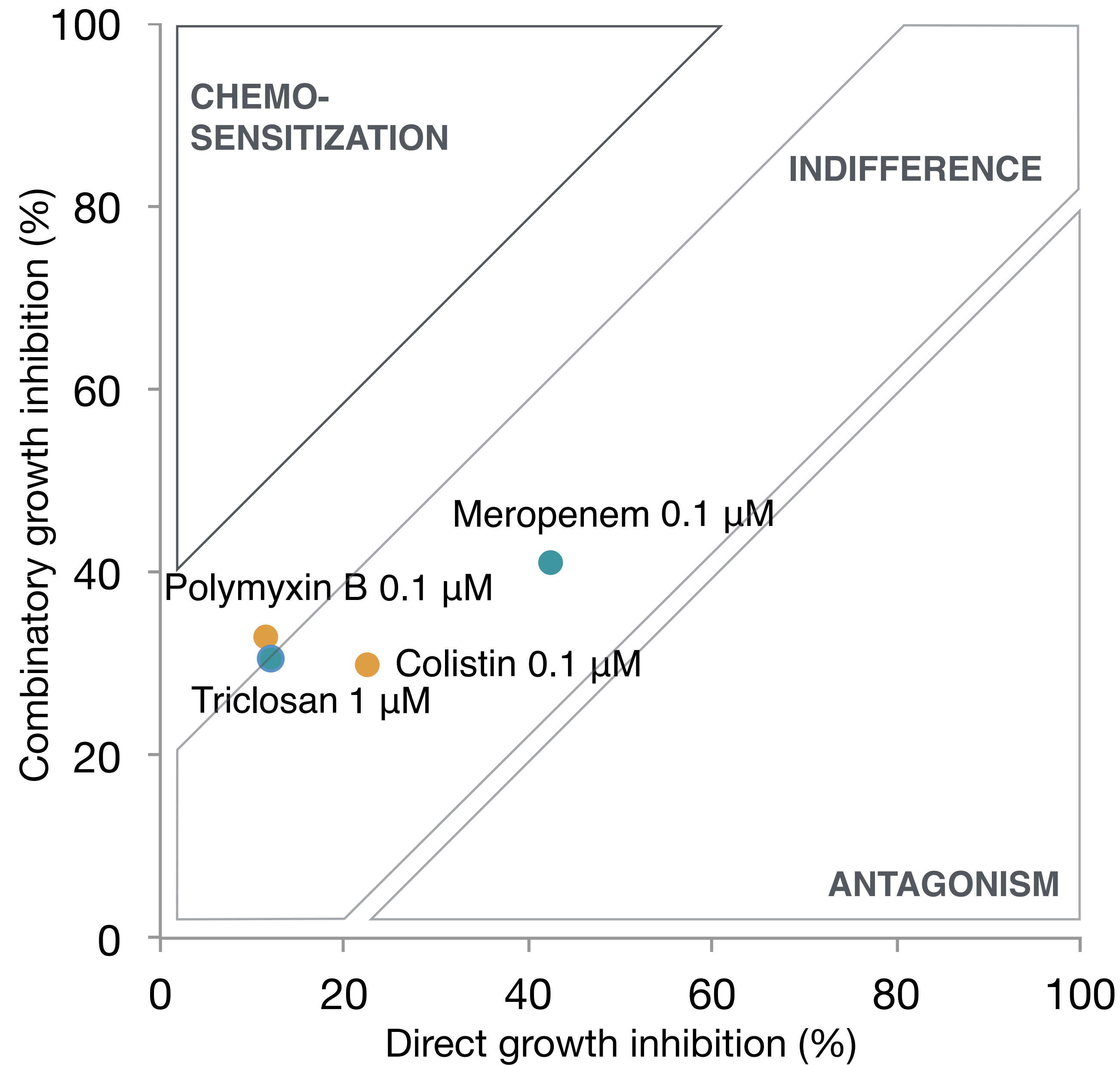

Supplement: FIGURE S7 — Monodose Chemosensitization retest. Above-MIC compounds were re-tested at 1 & 0.1μM. [file Image_7.PDF]
