# Supplementary material for: Multiparametric Profiling for Identification of Chemosensitizers against Gram-Negative Bacteria
Source: Front Microbiol. 2018 Feb 19;9:204. doi: 10.3389/fmicb.2018.00204 (PMC5845390; doi:10.3389/fmicb.2018.00204)
Supplement: FIGURE S8 — Validation of control chemicals mode of action. Real-time assays results for selectied hits were evaluated in a binary. [file Image_8.PDF]

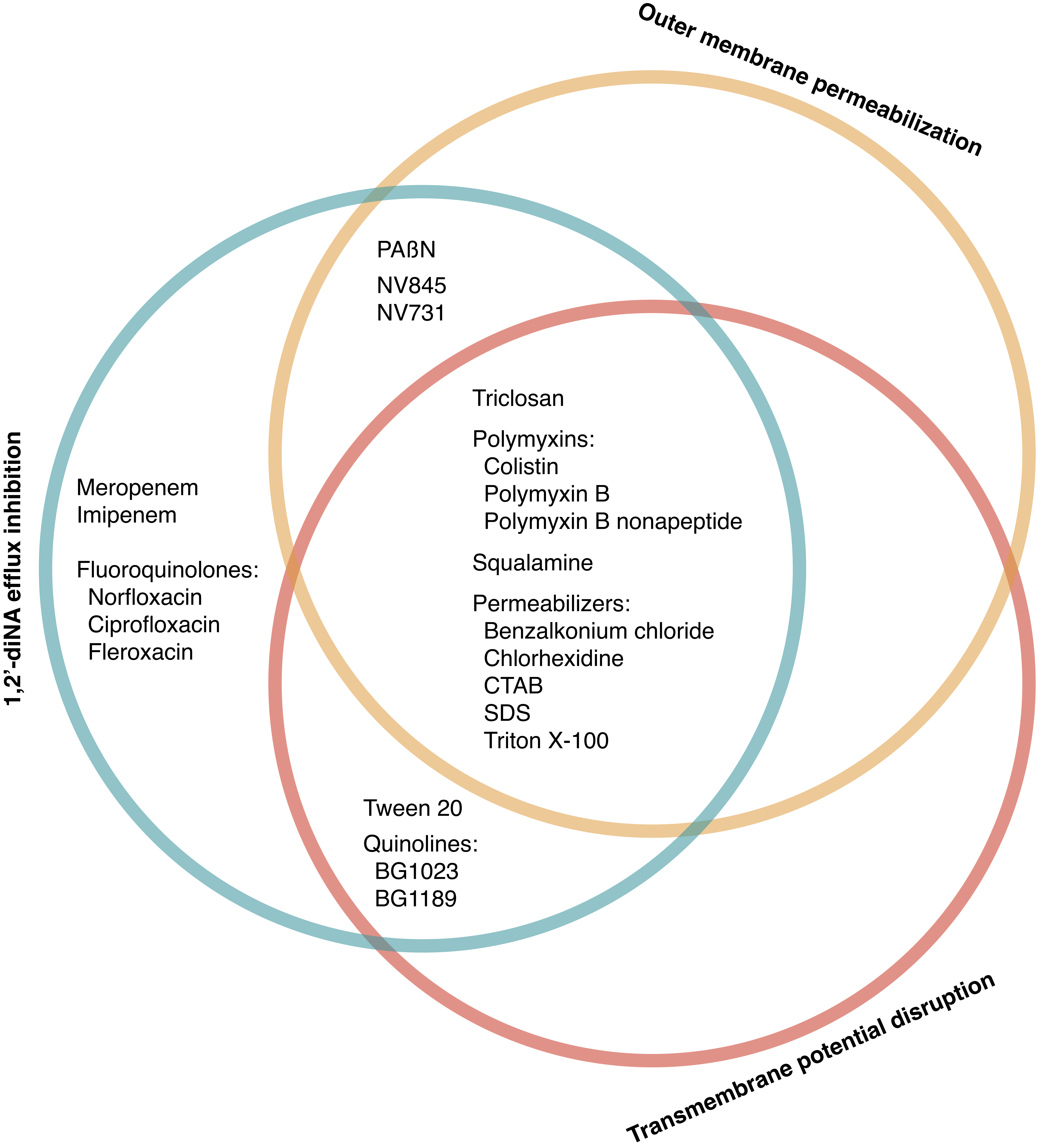
Outer membrane permeabilization
1,2'-diNA efflux inhibition
Transmembrane potential disruption
PAßN
NV845
NV731
Triclosan
Polymyxins:
Colistin
Polymyxin B
Polymyxin B nonapeptide
Squalamine
Permeabilizers:
Benzalkonium chloride
Chlorhexidine
CTAB
SDS
Triton X-100
Meropenem
Imipenem
Fluoroquinolones:
Norfloxacin
Ciprofloxacin
Fleroxacin
Tween 20
Quinolines:
BG1023
BG1189
