# Supplementary material for: Multiparametric Profiling for Identification of Chemosensitizers against Gram-Negative Bacteria
Source: Front Microbiol. 2018 Feb 19;9:204. doi: 10.3389/fmicb.2018.00204 (PMC5845390; doi:10.3389/fmicb.2018.00204)
Supplement: TABLE S1 — Components of the control chemicals library. [file Table_1.PDF]

Natural compounds

- eugenol
- thymol
- carnosic acid
- quercetin
- carvacrol
- deoxycholic acid
- taurocholic acid
- proflavine

Others

- lansoprazole
- omeprazole
- benzoic acid
- dinitrophenol
- formaldehyde
- ethanol
- salicylate
- reserpine
- sodium iodoacetate
- ethylenediaminetetraacetic acid (EDTA)
- ammonium persulfate
- sodium azide
- acetic acid
- glucose
- mannitol
- methyl viologen
- urea
- naphthylmethyl piperazine (NMP)
- verapamil
- procaine
- acriflavine
- triclosan
- carbonyl cyanide 3-chlorophenylhydrazone (CCCP)
- Phe-Arg β-naphthylamide (PAßN)

Phenothiazines

- thioridazine
- chlorpromazine

Polymyxins

- colistin
- polymyxin b
- polymyxin B nonapeptide

Polyamines

- squalamine
- NV845 : {3-[(3-Aminopropyl)methylamino]propyl}-((2E,6E)-3,7,11-trimethyldodeca-2,6,10-trienyl)amine **(1)**
- NV731 : {3-[Bis-(3-aminopropyl)amino]propyl}-(3,7-dimethylocta-2,6-dienyl)amine **(1)**

Permeabilizers

- benzalkonium chloride
- chlorhexidine
- cetyltrimethylammonium bromide (CTAB)
- tween 20
- sodium dodecyl sulfate (SDS)
- trimethylpentanylphenoxy ethanol (triton X-100)

Quinolines

- BG1023 : N1-(7-chloroquinolin-4-yl)-N2,N2-diisopropylethane-1,2-diamine **(2)**
- BG1189 : 3-(3-Dimethylaminopropyl)-6-nitro-3H-quinazolin-4-one **(3)**

Quinolones

- nalidixic acid
- norfloxacin
- ciprofloxacin
- fleroxacin

ß-lactams

- cefepime
- ceftazidime
- aztreonam
- ticarcillin
- cloxacillin
- piperacillin
- ertapenem
- meropenem
- imipenem

Cyclines

- doxycycline
- tetracycline
- tigecycline

Aminoglycosides

- kanamycin
- tobramycin
- gentamicin

Other antibiotics

- rifampicin
- tylosin
- novobiocin
- erythromycin
- sulfamethoxazole
- bacitracin a
- tazobactam
- clavulanate
- florfenicol
- chloramphenicol

1- Berti, L., Bolla, JM., Brunel, JM., Casanova, J., Lorenzi, V. (2012).Use of polyaminoisoprenyl derivatives in antibiotic or antiseptic treatment. Patent No :WO2012113891 A1.

2- Ghisalberti, D., Mahamoud, A., Chevalier, J., Baitiche, M., Martino, M., Pagès, JM. et al. (2006).Chloroquinolines block antibiotic efflux pumps in antibiotic-resistant Enterobacter aerogenes isolates. Int J Antimicrob Agents. 27, 565–569. doi:10.1016/j.ijantimicag.2006.03.010.

3- Chevalier, J., Mahamoud, A., Baitiche, M., Adam, E., Viveiros, M., Smarandache, A.,et al. (2010). Quinazoline derivatives are efficient chemosensitizers of antibiotic activity in Enterobacter aerogenes, Klebsiella pneumoniae and Pseudomonas aeruginosa resistant strains. Int J Antimicrob Agents. 36, 164–168. doi:10.1016/j.ijantimicag.2010.03.027.
