# Supplementary material for: Multiparametric Profiling for Identification of Chemosensitizers against Gram-Negative Bacteria
Source: Front Microbiol. 2018 Feb 19;9:204. doi: 10.3389/fmicb.2018.00204 (PMC5845390; doi:10.3389/fmicb.2018.00204)
Supplement: TABLE S2 — Transmembrane potential disruption assay. Showed transmembrane potential disruption (+), did not show transmembrane potential disruption (-), the measurement could not be assessed due to strong fluorescence quenching (q). [file Table_2.PDF]

Hit	Transmembrane potential disruption
Triclosan	+
CCCP	q
PAßN	-
Thioridazine	q
Chlorpromazine	q
Colistin	+
Polymyxin B	+
PMB nonapeptide	+
Squalamine	+
NV845	-
NV731	-
Benzalkonium Chloride	+
Chlorhexidine	+
CTAB	+
Tween 20	+
SDS	+
Triton X-100	+
BG1023	+
BG1189	+
Fleroxacin	-
Ciprofloxacin	-
Norfloxacin	-
Imipenem	-
Meropenem	-
