# Supplementary material for: Multiparametric Profiling for Identification of Chemosensitizers against Gram-Negative Bacteria
Source: Front Microbiol. 2018 Feb 19;9:204. doi: 10.3389/fmicb.2018.00204 (PMC5845390; doi:10.3389/fmicb.2018.00204)
Supplement: TABLE S3 — Relationship between %OPS, %EIS and %MCS by linear regression analysis. The relationship between chemosensitization and barriers to be altered was modeled in a multiple linear regression analysis. %MCS was hypothesized to be dependent on %OPS or %EIS and the analysis was performed on the previously selected hits except thioridazine and chlorpromazine as EIS could not be calculated. The full data set comprised %EIS, %OPS and %MCS for the 22 selected compounds. The MCS was calculated after retest at lower concentration as indicated previously for colistin, polymyxin B, meropenem and triclosan. [file Table_3.PDF]

Linear Regression

Regression Statistics

R	0,722
R Square	0,522
Adjusted R Square	0,498
S	21,755
Total number of observations	22,00

MCS = 0,3268 * OPS + 0,2152 * EIS

ANOVA

	d.f.	*SS*	*MS*	*F*	*p-level*
Regression	2,	10 317,865	5 158,932	10,901	0,001
Residual	20,	9 465,295	473,265		
Total	22,	19 783,16			

	Coefficients	*Standard Error*	*LCL*	*UCL*	*t Stat*	*p-level*	*H0 (5%) rejected?*
**Intercept**	0,00						
**OPS**	0,327	0,163	-0,012	0,666	2,01	0,058	*No*
**EIS**	0,215	0,19	-0,182	0,612	1,131	0,272	*No*
T (5%)	2,086						

LCL - Lower value of a reliable interval (LCL)
UCL - Upper value of a reliable interval (UCL)
